# Evolution of Hepatic Steatosis to Fibrosis and Adenoma Formation in Liver-Specific Growth Hormone Receptor Knockout Mice

**DOI:** 10.3389/fendo.2014.00218

**Published:** 2014-12-18

**Authors:** Yong Fan, Xin Fang, Asako Tajima, Xuehui Geng, Sarangarajan Ranganathan, Henry Dong, Massimo Trucco, Mark A. Sperling

**Affiliations:** ^1^Institute of Cellular Therapeutics, Allegheny Health Network, Pittsburgh, PA, USA; ^2^Division of Immunogenetics, Department of Pediatrics, University of Pittsburgh School of Medicine, Pittsburgh, PA, USA; ^3^Department of Pediatrics, Fujian Medical University Union Hospital, Fuzhou, China; ^4^Department of Pathology, Children’s Hospital of Pittsburgh of UPMC, Pittsburgh, PA, USA; ^5^Division of Endocrinology, Department of Pediatrics, University of Pittsburgh School of Medicine, Pittsburgh, PA, USA

**Keywords:** growth hormone receptor, hepatic steatosis, hepatocellular adenoma, NAFLD

## Abstract

**Background:** Non-alcoholic fatty liver disease (NAFLD) is one of the most common forms of chronic liver diseases closely associated with obesity and insulin resistance; deficient growth hormone (GH) action in liver has been implicated as a mechanism. Here, we investigated the evolution of NAFLD in aged mice with liver-specific GHR deletion.

**Methods:** We examined glucose tolerance, insulin responsiveness, and lipid profiles in aged male mice (44–50 weeks) with GHRLD. We performed proteomics analysis, pathway-based Superarray assay, as well as quantitative RT-PCR to gain molecular insight into the mechanism(s) of GHR-deficiency-mediated NAFLD. In addition, we examined the pathological changes of livers of aged GHRLD male mice.

**Results:** The biochemical profile was consistent with that of the metabolic syndrome: abnormal glucose tolerance, impaired insulin secretion, and hyperlipidemia. RT-qPCR analysis of key markers of inflammation revealed a three- to fivefold increase in TNFα and CCL3, confirming the presence of inflammation. Expression of fibrotic markers (e.g., Col1A2 and Col3A1) was significantly increased, together with a two- to threefold increase in TGFβ transcripts. Proteomics analyses showed a marked decrease of Mup1 and Selenbp2. In addition, pathway-analysis showed that the expression of cell cycle and growth relevant genes (i.e., Ccnd1, Socs2, Socs3, and Egfr) were markedly affected in GHRLD liver. Microscopic analyses (H&E) of GHRLD livers revealed the presence of hepatic adenomas of different stages of malignancy.

**Conclusion:** Abrogation of GH signaling in male liver leads to metabolic syndrome, hepatic steatosis, increased inflammation and fibrosis, and development of hepatic tumor. Since obesity, a common precursor of NAFLD, is a state of deficient GH secretion and action, the GHRLD model could be used to unravel the contribution of compromised hepatic GH signaling in these pathological processes, and help to identify potential targets for intervention.

## Introduction

Non-alcoholic fatty liver disease (NAFLD) is the most common type of liver disease that affects 20–40% of the adult population in developed countries. It describes a spectrum of conditions of hepatic fat accumulation that is not caused by excessive alcohol consumption, but rather closely associated with metabolic disorders (1). While the underlying mechanisms of NAFLD are not fully understood at present, factors relevant to metabolic syndromes, such as insulin resistance, obesity, hyperlipidemia, and hyperglycemia, all contribute to excessive fat deposition in the liver (2–4). Left untreated, NAFLD can progress to NASH (non-alcoholic steatohepatitis), where lipid accumulation in hepatocytes leads to cellular damage and chronic inflammation, resulting in liver fibrosis and cirrhosis, as well as the development of hepatic carcinomas and other forms of liver tumors (5, 6).

Growth hormone (GH), produced and secreted by the pituitary gland, is a pleiotropic hormone that functions as a key regulator of postnatal body growth and development. It controls a broad range of anabolic processes, such as cellular proliferation, differentiation, nitrogen retention, and bone elongation. In addition, evidence has accumulated for a role of GH in maintaining metabolic homeostasis in adults. Mice deficient in its cognate receptor, the growth hormone receptor (GHR), have increased adiposity while exhibiting improved insulin sensitivity (7). Abrogating GHR specifically in β-cells results in impaired glucose stimulated insulin secretion and decreased compensatory proliferation in response to high fat diet (8). While the role of GH signaling in muscle cells remains under debate, both insulin sensitivity and adiposity are affected in GHR muscle-specific knockout mice (9, 10). Deletion of GHR in fat cells results in increased body fat accumulation, with no detectable influence on glucose metabolism (11, 12).

To study the function of GH signaling in liver, we previously generated mice with GHR liver-specific deletion (GHRLD) (13). GHRLD mice develop hyperglycemia, hyperinsulinemia, and marked insulin resistance as a result of high circulating GH acting on tissues such as muscle and fat in which GH signaling has remained intact (13). Notably, GHRLD mice develop severe hepatic steatosis as early as 6–8 weeks of life while on a normal diet, largely due to increased triglyceride (TG) synthesis together with compromised export of TG and elevated supply of free fatty acids (13, 14). These findings have been confirmed in a separate independent study (15). Consistent with these findings, abrogation of signal transducer and activator of transcription 5 (STAT5) (16, 17), or Janus kinase 2 (JAK2) (14), the downstream signaling cascade of GH mediated through GHR in liver, results in hepatic steatosis and enhanced cell proliferation. Thus, GH and its downstream signaling, mediated through GHR, are essential for normal hepatic lipid metabolism and homeostasis.

In the present study, we investigated NAFLD progression in aged GHRLD mice (44–50 weeks) and found that a significant percentage developed symptoms of NASH, with increased markers of inflammation and fibrosis. Moreover, a subset of GHRLD mice ultimately developed hepatic adenomas spontaneously, while on a normal chow diet.

## Materials and Methods

### Mice

C57BL/6 mice were purchased from the Jackson Laboratory (Bar Harbor, ME, USA). GHR liver-specific deletion (B6.GHRLD) mice have been described previously (13). Unless specified otherwise, only male GHRLD mice and age- and gender-matched controls were used. All mice were housed in a specific pathogen-free animal facility at the Rangos Research Center, Children’s Hospital of Pittsburgh. All animal experiments were carried out under protocols approved by the Institutional Animal Care and Usage Committee of the University of Pittsburgh.

### RT-qPCR analysis

The total RNA of a liver sample was isolated using RNeasy mini kit, according to the manufacturer’s protocol (#74104, Qiagen, Valencia, CA, USA). Following DNase I treatment (Ambion, Life Technologies, Grand Island, NY, USA), RNA samples were reverse-transcribed into cDNAs with Superscript III cDNA kit (#18080-051, Invitrogen). qPCR analyses of gene expression in cDNA samples were performed with the LightCycler FastStart DNA Master SYBR Green I kit, and analyzed with the LightCycler 2 software (#03003230001, Roche Applied Science), as previously described (18). The following primers were used: Hprt (F 5′-GGATACAGGCCAGACTTTGTTGGA-3′, R 5′-CAACAGGACTCCTCGTATTTG CAG-3′), Col1A2 (F 5′-CCAGAGTGGAACAGCGATTAC-3′, R 5′-GATGCAGGTTTCACCA GTAGAG-3′), Col3A1 (F 5′-CCTGGTGGAAAGGGTGAAAT-3′, R 5′-CGTGTTCCGGGTATAC CATTAG, Col6A1 (F 5′-ACGTGTTTGACTTCATCCCAGGCT-3′, R 5′-AGATCTGGGCGGTGACATTCTTCA-3′), TGFβ1 (F 5′-CGAAGCGGACTACTATGCT AAA-3′, R 5′-TCCCGAATGTCTGACGTATTG-3′), CCL3 (F 5′-GAAGATTCCACGCCAATTC ATC-3′, R 5′-GATCTGCCGGTTTCTCTTAGTC-3′), IL-1a (F 5′-GAAGAAGAGACGGCTGAGT TT-3′, R 5′-TCACTCTGGTAGGTGTAAGGT-3′), IL-1b (F 5′-CCACCTCAATGGACAGAATAT CA-3′, R 5′-CCCAAGGCCACAGGTATTT-3′), TNFα (F 5′-TTGCTCTGTGAAGGGAATGG-3′, R 5′-GGCTCTGAGGAGTAGACAATAAAG-3′), Cse3a (F 5′-TTCGGTAACTCTGCTGGAGGC ATT-3′, R 5′-ACTCTGCGATATGGCTCTGTGGAA-3′), Selenbp2 (F 5′-AAGGGCTGGATGTTG CCAGAAATG, R 5′-TGCAGCCAGTTGCTGAAGTAAAGG-3′), Mup1 (F 5′-ATGAAGATGCT GCTGCTGCTG-3′, R 5′-ATTCTTCATTCTCGGGCCTG-3′). Ccnd1 (F 5′-CAGAGGCGGATGAGAACAAG-3′, R 5′-GAGGGTGGGTTGGAAATGAA-3′).

Superarray analysis was performed with ABI 7900HT real-time PCR system (Applied Biosystems). Total RNA samples were isolated from 8 to 10 weeks old male GHRLD and controls, and reverse-transcribed as described above. cDNA samples were subjected to assay with mouse JAK/STAT Signaling Pathway RT^2^ *Profiler*™ PCR Array kit (Qiagen), following the manufacturer’s instructions. The Superarray experiment was performed in triplicate. Relative levels of gene expression were determined by the data analyzer template provided by Qiagen (http://www.qiagen.com/us/products/genesandpathways/data-analysis-center-overview-page).

For RT-PCR analysis of Ccnd1 mRNA expression, 2 μg of total RNA isolated from 44 to 50 weeks old male GHRLD (*n* = 4) and control mice (*n* = 4) were reversely transcribed as above. cDNA samples transcribed from 10 ng of total RNA were subjected to PCR amplification, with primers specific to Ccnd1 (30 cycles) and Hprt (25 cycles), and separated by agarose gel electrophoresis.

### Blood glucose measurement and intraperitoneal glucose tolerance test

Blood glucose levels were measured with the Ascensia Contour blood glucose monitoring system (Bayer HealthCare LLC, Mishawaka, IN, USA). IPGTT was performed as previously described (19). Briefly, mice were fasted overnight (~16 h) and injected intra-peritoneally with a bolus of 2 g of d-glucose (Sigma-Aldrich, St. Louis, MI, USA) per kilogram of body weight. Blood was sampled from a small nick of the tail-vein at 0, 15, 30, 60, 90, and 120 min after glucose injection.

### Liver histology

Liver samples were preserved in 10% phosphate buffered formalin (Fisher Scientific). Fixed liver specimens were embedded in paraffin, sectioned with microtome (Leica Microsystems). Five micron sections were stained with hematoxylin and eosin (H&E), and examined by a pathologist specialized in liver tumors at Children’s Hospital of Pittsburgh. Tumor types were scored according to the WHO international classification of rodent tumors (20).

### Liquid chromatography fractionation of lipoproteins

Plasma samples were harvested and pooled from 16 to 20 weeks old male GHRLD mice (*n* = 6) and age- and gender-matched control littermates (*n* = 6). Two hundred fifty microliters of plasma samples were loaded to Superose 6 10/300GL high-performance Tricorn columns (GE Healthcare Life Sciences) using a fast protein liquid chromatography system (Amersham Biosciences), and eluted with PBS at a constant flow rate of 0.5 ml/min. TG and cholesterol concentrations in each fraction (500 μl) were assayed with Thermo Infinity TG and Thermo Infinity cholesterol kits, respectively (Thermo Scientific).

### Western blot and ELISA

Protein extracts were isolated from homogenized liver tissues of 16–20 weeks male GHRLD mice and littermate controls, using T-PER Tissue Protein Extraction Reagent (#78510, Pierce Biotechnology, Rockford, IL, USA), following manufacturer’s suggested protocol. Twenty micrograms of protein lysates were separated with electrophoresis, transferred to PVDF membrane, and probed with primary antibodies against Ces3 (1:1000, #AF5985, R&D Systems, Minneapolis, MN, USA) and Selenbp2 (1:1000, #orb4825, Biorbyt LLC, San Francisco, CA, USA). The membranes were subsequently blotted with HRP-conjugated secondary antibodies, and incubated with ECL plus chemiluminescent substrates (Pierce Biotechnologies, Thermo Scientific). The intensity of protein bands was quantified by densitometry using the ImageJ software (NIH, Bethesda, MD, USA).

Levels of Mup1 expression in livers were analyzed with the Mouse Major Urinary Protein-1 Immunoassay Kit (#orb54814, Biorbyt LLC, San Francisco, CA, USA), following manufacturer’s protocol. To ensure that the Mup1 concentrations in the samples were within the detection range of the assay kit, liver lysates of controls and GHRLD mice were diluted 2000- and 500-fold in 1× assay buffer, respectively. The results were normalized to the concentration of proteins in the lysates, measured by BCA assay kit (#23225, Pierce Biotechnologies, Thermo Scientific), following manufacturer’s protocols.

### Proteomics analysis

Proteomics analyses of liver samples were described previously (21). Briefly, liver tissues (40 mg) were homogenized in 800 μl of Mammalian Protein Extraction Reagent (M-PER) buffer supplemented with protease inhibitor cocktail (Pierce Biotechnology, Rockford, IL, USA). Albumin was removed from protein extracts with Aurum serum protein mini kit (Bio-Rad Laboratory, Inc.). Three hundred micrograms of liver protein samples of GHRLD and control mice were precipitated by 2-D Clean-Up Kit (GE Healthcare, Piscataway, NJ, USA) and dissolved in 90 μl lysis buffer (7 mol/l urea, 2 mol/l thiourea, 4% w/v CHAPS, 1% v/v Triton X-100, 10 mmol/l dithiothreitol). Thirty microliters of each sample was combined to create a mixed standard sample for Cy2 labeling. The remaining aliquots of the control and GHRLD samples were incubated with 1 nmol Cy3 or 1 nmol Cy5, respectively. After quenching, the samples were mixed with immobilized pH gradient (IPG) buffer, and applied to an IPG strip (pH 4–7, 24 cm) and incubated for 20 h using low voltage (30 V) in an Ettan IPGphor II IEF system (GE Healthcare). After isoelectric focusing (300 V for 30 min, 500 V for 30 min, 1000 V for 1 h, and 8000 V for 10 h), the strip was equilibrated with 10 ml of 2.5% w/v iodoacetamide containing equilibration buffer [2% w/v sodium dodecyl sulfate (SDS), 50 mmol/l Tris–HCl pH 8.8, 6 mol/l urea, 30% glycerol, and 0.001% bromophenol blue]. Second-dimension SDS–polyacrylamide gel electrophoresis was performed by transferring the IPG strip to a 12.5% single-percentage gel and electrophoresing for about 18 h at 10°C.

Two-dimensional (2-D) gels were scanned using a Typhoon 9400 variable mode imager (GE Healthcare). Imager settings used blue-excited fluorescence (488 nm) for Cy2, green-excited fluorescence (532 nm) for Cy3, and red-excited fluorescence (633 nm) for Cy5. Data analysis was performed using DeCyder differential analysis software, version 5.02 (GE Healthcare). Gel images were processed for spot detection and determination of the relative protein abundance based on fluorescence intensity, defined as spot volume. Change of expression of a specific protein was determined by dividing the spot volume in the GHRLD sample by that of the control sample. Protein spots were selected as up-regulated or down-regulated among those exceeding a twofold difference in fluorescence intensity. Differentially expressed proteins were manually spot-picked from Coomassie Blue G-250 (Bio-Rad, Hercules, CA, USA) stained gels.

Dried peptides from in-gel digestion were dissolved in 3 μl of 50% acetonitrile and 0.3% TFA, and mixed with 3 μl of freshly prepared matrix solution (10 mg/ml α-cyano-4-hydroxy-cinnamic acid in 50% acetonitrile, 0.3% TFA). The mixture, 0.6 μl, was spotted onto a MALDI plate (Applied Biosystems). The 4700 Proteomics Analyzer MALDI-TOF/TOF (Applied Biosystems) was used to identify proteins from the trypsin digest. Analysis of samples used reflector positive ion mode acquisition and processing method to collect peptide spectra in the mass range of 800–4000 days. The 10 highest-intensity peptides were selected for tandem mass spectrometry analysis using tandem mass spectrometry mode acquisition with the 1-kV positive ion and processing method. Data processing was performed with GPS Explorer Workstation (Applied Biosystems) and MASCOT database analysis of mammalian proteins.

### Statistical analysis

All values are expressed as the mean ± SEM unless otherwise specified. Statistical significance was determined using non-parametric Mann–Whitney *t*-test, unless otherwise specified. All statistical analyses were carried out with the GraphPad Prism 4.0 Software. In all experiments, differences were considered significant when *p* was <0.05.

## Results

### Development of metabolic disorders in aged GHRLD mice

**Figure 1:**
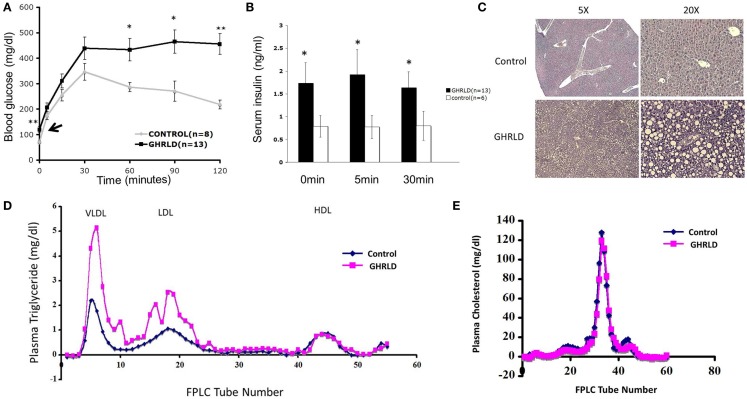
**Aged GHRLD mice developed glucose intolerance, hyperlipidemia, and hepatic steatosis**. **(A)** Intraperitoneal glucose tolerance test of 44–50 weeks old GHRLD mice (*n* = 13) and age-matched controls (*n* = 8). Arrow indicates the blood glucose levels of GHRLD mice after overnight fasting (16 h). Data are presented as mean ± SEM. *p < 0.05; **p < 0.01. **(B)** Serum insulin levels in GHRLD (filled bars, *n* = 13), or control (open bars, *n* = 6) mice. Sera were collected from either after overnight fasting (0 min), 5- or 60-min after i.p. injection of a bolus of 2 g/kg of d-glucose. Data are presented as mean ± SEM. *p < 0.05. **(C)** Representative histological sections (H&E) of liver samples harvested from 44 to 50 weeks old GHRLD (lower panels) and age-matched controls mice (upper panels). Left panels, 5× objective lens magnification; right panels, 20×. **(D,E)** Effect of liver-specific GHR deletion on lipoprotein metabolism. Aliquots of plasma (250 μl) pooled from individual mice in GHRLD mice (*n* = 6) and control mice (*n* = 6) subjected to gel filtration chromatography, followed by the determination of TG **(D)** and cholesterol **(E)** concentrations in fractions. The peak fractions of 3–10, 14–23, and 40–50 in **(D)** represent VLDL, LDL, and HDL, respectively.

We have shown previously that liver-specific deletion of GHR result in lipid accumulation in hepatocytes and insulin resistance (13). To investigate the long-term pathological impact of GH-signaling deficiency, we examined the fasting blood glucose levels of aged GHRLD mice (44–50 weeks old). Elevated levels of both fasting blood glucose (Figure 1A, black arrow) and plasma insulin (Figure 1B) were observed. In addition, aged GHRLD mice exhibited abnormalities in maintaining blood glucose homeostasis upon intraperitoneal glucose challenge (IPGTT, Figure 1A), as well as hyperinsulinemia (Figure 1B). Histological sections of liver tissues harvested from GHRLD mice revealed severe steatosis, in line with our previous findings on GHRLD mice of 8–16 weeks old (Figure 1C).

GHRLD mice display elevated levels of plasma TG, increased liver influx of free fatty acid, and markedly reduced VLDL–TG liver output (13). To further understand the association of liver GH signaling with lipid metabolism, we subjected plasma samples pooled from individual GHRLD mouse to gel filtration column chromatography and examined the TG composition in each lipoprotein fractions. Striking differences of TG lipoprotein profile were observed between GHRLD and control samples (Figure 1D): GHRLD plasma samples exhibited marked increased levels of VLDL–TG and LDL–TG, whereas HDL—TG levels were similar to controls. No significant difference of cholesterol levels was observed between GHRLD and control mice (Figure 1E). Taken together, these results suggested that GH signaling deficiency in liver leads to hyperlipidemia and other features of the metabolic syndrome.

### Proteomic analysis of effects of GH-signaling deficiency in liver

**Figure 2:**
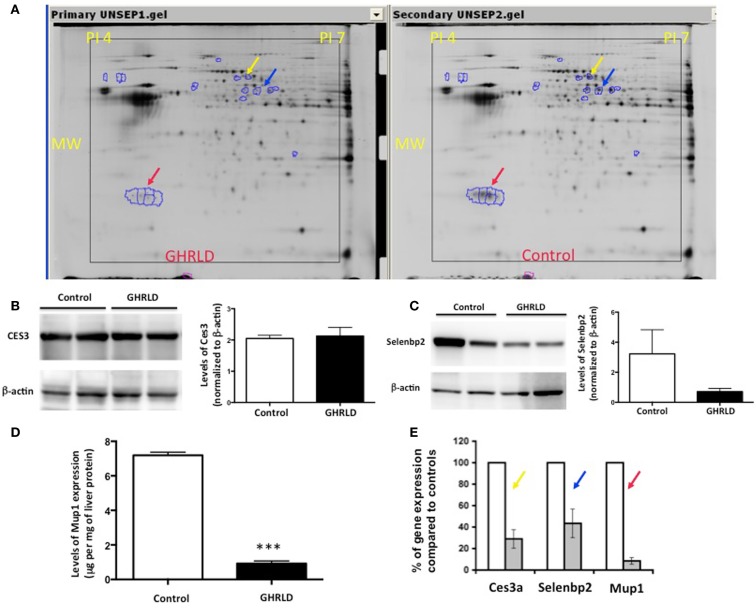
**Proteomic analysis of effects of liver-specific GHR deletion**. **(A)** Proteins from 8- to 10-week old male GHRLD liver samples (*n* = 4, left panel) were extracted and compared to samples harvested from control littermates (*n* = 4, right panel) on 2-D electrophoresis (Isoelectric point pH 4–7). Arrows indicate proteins that were down-regulated in GHRLD liver, which were further identified by mass spectrum. Red arrow, major urinary protein-1 (Mup1); blue arrow, selenium binding protein 2 (Selenbp2); yellow arrow, Caboxylesterase 3A (Cse3a or Es31). MW, molecular weight. **(B,C)** *Left* panels, Western blot analyses of Cse3a **(B)** and Selenbp2 **(C)** expression levels; *Right* panels, semi-quantification of Western blot results. **(D)** Levels of Mup1 in liver lysates of GHRLD (*n* = 3) and control (*n* = 3) mice. ***p < 0.001. **(E)** RT-qPCR analysis of mRNA expression of genes identified by proteomics in GHRLD liver samples (*n* = 4, gray bar), normalized to controls (*n* = 4, open bar).

To gain molecular insight of GH-signaling deficiency-mediated hepatic steatosis, we harvested livers from 8- to 10-week male GHRLD mice and age- and gender-matched controls and subjected these samples to MALDI-based proteomic analysis. As shown in Figure 2A, three protein spots/clusters whose expression levels were markedly altered (>2-fold) were identified (Figure 2A, arrows). These were major urinary protein-1 (Mup1), carboxylesterase 3A (Ces3a or Es31), and Selenium binding protein 2 (Selenbp 2). Mup1 is a secreted protein of the lipocalin family, which forms complexes with pheromones in circulation and excreted in urine to mediate chemical communication between rodents. Recent studies have suggested additional roles of Mup1 in regulating metabolic homeostasis, by inhibiting the expression of both gluconeogenic and lipogenic genes in the liver (22). While the roles of Ces3a and Selenbp 2 in lipid metabolism remains unknown, the TG hydrolase activity of other carboxylesterase family members (e.g., Ces1) has been shown to regulate hepatic lipid biosynthesis, secretion, deposition, and fatty acid oxidation in liver (23, 24). To demonstrate that the observed differential protein expression on the 2-D gel indeed reflected changes in protein translation levels, we performed Western blot analyses of Ces3a and Selenbp 2 in liver samples of GHRLD mice (Figures 2B,C). Consistent with results from the 2-D gel, marked decrease of Selenbp 2 protein expression levels was observed (Figure 2C). However, no significant change of Ces3a was detected (Figure 2C). The discrepancy between 2-D gel and Western blot results might be due to either changes in post-translational modifications (e.g., acetylation or phosphorylation), or cross reactivity of the primary antibody to other isoforms of the Ces3 (e.g., Ces3b). The levels of Mup1 expression in male GHRLD livers were also significantly decreased, as demonstrated with ELISA (Figure 2D). RT-qPCR analysis revealed significant decrease of mRNA transcript levels of all three genes in male GHRLD liver (Figure 2E), suggesting that GH-signaling regulates the expression of these genes at transcription levels.

### Impact of GH-signaling deficiency on JAK/STAT pathway

**Figure 3:**
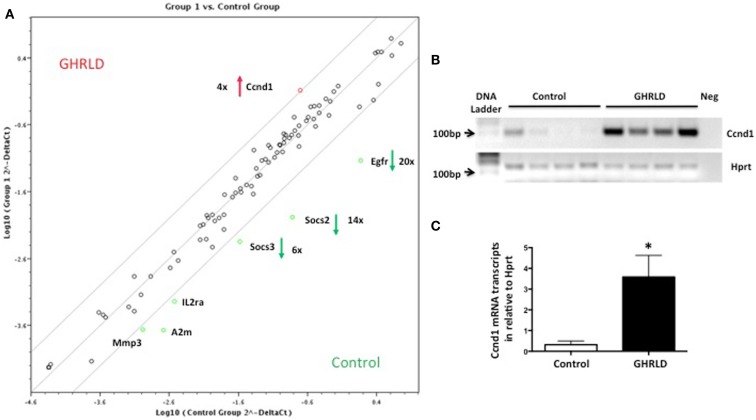
**Superarray analysis of effects of GH-signaling deficiency on the expression of genes related to the STAT/JAK signaling pathway**. **(A)** Total RNA samples from 8 to 10 weeks old male GHRLD livers (*n* = 6) were extracted and pooled, and were subjected to RT-qPCR based Superarray analyses in triplicate. Levels of gene expression were normalized to a composite group of house-keeping genes, and compared to those of age-matched control samples (*n* = 6). Shown is the scatter plot comparing the levels of gene expression between GHRLD mice (*y*-axis) and controls (*x*-axis). Among the 84 genes analyzed, only 4 highly expressed genes (Ccnd1, Egfr, Socs2, and Socs3) exhibited >4-fold alteration. The numbers represent the folds of changes of gene expression, in comparison to controls. Red arrow and green arrows indicate increase and decrease of gene expression in the GHRLD samples, respectively. **(B)** RT-PCR analysis of Ccnd1 mRNA expression in livers of 44–50 weeks old, male GHRLD (*n* = 4) and control mice (*n* = 4). Neg, PCR reaction without cDNA input. **(C)** RT-qPCR analysis of Ccnd1 mRNA expression in GHRLD liver samples (*n* = 4), in comparison with controls (*n* = 4). *p < 0.05.

Liver-specific abrogation of both GHR and STAT5 results in hepatic steatosis. To further understand the underlying molecular mechanism, we performed RT-qPCR based Superarray analyses to examine changes of the mRNA transcription of genes downstream of the STAT/JAK pathway. Among all the four genes that were found to be altered >4-fold, Socs2, Socs3, and Egfr were down-regulated, whereas Cyclin D1 (Ccnd1) was found to be up-regulated (Figure 3A). Genes at the lower left corner (Mmp3, A2m, and IL2a) with crossing points >25 (30, 29, and 28, respectively) were excluded for further analysis as only background levels of expression was detected. While acting as a key regulator of cell-cycle in tissues with rapid renewal, Ccnd1 is expressed in the liver at its quiescent stage, and has recently been implicated in regulating the expression of gluconeogenic genes, via, in part, inhibiting peroxisome proliferator-activated receptor γ coactivator-1α (PGC1α) activity (25, 26). Both SOCS2 and SOCS3, members of suppressor of cytokine signaling (SOCS) family, are negative regulators of the JAK/STAT signaling pathway. SOCS2 has recently been implicated in regulating fat metabolism (27). While the metabolic role of EGFR is largely unknown, EGFR was shown as a critical regulator of hepatocyte proliferation for efficient liver regeneration (28). These data suggest that GH-signaling deficiency has broad impacts on various downstream events that are essential for liver homeostasis.

### Impact of GH-signaling deficiency on the expression of fibrotic and inflammatory genes

**Figure 4:**
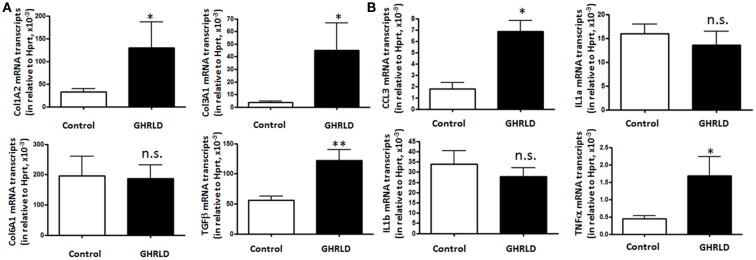
**Effects of GHR deletion on fibrotic and inflammatory gene expression**. Liver tissues collected from 44 to 50 weeks old male GHRLD mice (*n* = 9) and age-matched controls (*n* = 6) were used for the preparation of total RNA. Hepatic mRNA levels of genes relevant to fibrosis **(A)** and inflammation **(B)** were determined using real-time quantitative RT-PCR assay. *p < 0.05, **p < 0.01, n.s. not significant.

Fat accumulation in the liver leads to increase of inflammation and fibrosis. To gain mechanistic insight into GH-signaling deficiency-mediated NAFLD, we examined the mRNA transcription of fibrotic genes (Figure 4A), as well as pro-inflammatory cytokine genes (Figure 4B) by RT-qPCR analyses. As shown in Figure 4A, the mRNA expression of both Col1A2 and Col3A1 were significantly elevated in GHRLD livers. In consistence, expression of TGF-β, a key regulator of the fibrotic response, was also increased (29). No difference of Col6A1 transcription was observed between GHRLD liver samples and controls.

Transcription of CCL3 [Chemokine C–C motif ligand 3, also known as macrophage inflammatory protein-1α (MIP-1α)], a cytokine that is involved in the recruitment and activation of neutrophils and other polymorphonuclear leukocytes in response to acute inflammation, was up-regulated. TNFα expression is also increased significantly, whereas levels of interleukin 1a and 1b (IL-1a and IL-1b) transcripts were not changed significantly (Figure 4B). These results suggested that long-term fat accumulation in GHRLD livers is associated with increased expression of fibrotic and inflammatory genes.

### Progression from NAFLD to hepatic tumor formation in aged GHRLD mice

**Figure 5:**
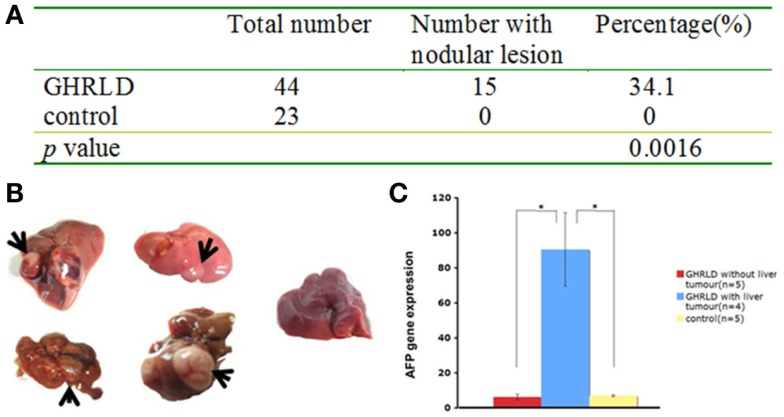
**Development of hepatic tumors in 50–70 weeks old GHRLD mice**. **(A)** Summary of hepatic tumor development in aged GHRLD mice. **(B)** Representative photographic images of livers of GHRLD (four on the left) and control (one on the right) livers. Arrows indicate the tumors. **(C)** RT-qPCR analysis of α-fetoprotein (AFP) mRNA expression, a marker for hepatocelluar adenomas. *p < 0.05.

We noticed that about 30–40% of livers in 55–70 weeks old GHRLD mice displayed nodule like structures, whereas none was observed in age-matched controls (Figures 5A,B). To validate that the nodules observed were of neoplastic nature, we performed RT-qPCR analysis of the levels of mRNA transcripts of *Afp*, the gene that encodes the α-fetoprotein (AFP), which is abundantly expressed in fetal liver, but silenced transcriptionally in adult. Elevated levels of AFP in adult were used as a biomarker for the development of liver tumors, such as hepatocellular carcinoma (HCC) and hepatic adenoma. As shown in Figure 5C, the levels of *Afp* mRNA transcripts in GHRLD livers with nodules were significantly higher than those of age-matched control livers, or GHRLD livers with no nodule formation (Figure 5C).

**Figure 6:**
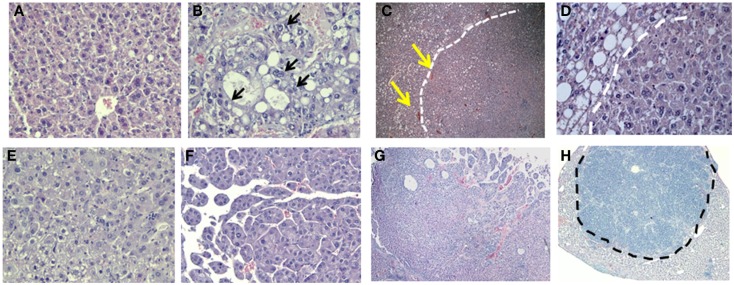
**Histological analysis (H&E) of hepatic adenomas in 50–70 weeks old GHRLD mice**. Representative images of liver sections (5 μm) of control [**(A)**, 200×] and GHRLD [**(B,E,F)**, 200×; **(C,G,H)**, 50×; **(D)**, 400×] mice. Arrows in **(B)** show the abnormal nuclear variations. While dotted lines in **(C,E)** show the boundaries between the tumor region and the adjacent hepatic steatotic region. Yellows arrows in **(C)** indicate the dilated blood vessels at the edges of the nodular structure. **(E)** shows typical images of benign hepatic adenoma, similar to HNF1α mutated type. **(F**,**G)** show regions of atypical hepatic adenomas with macrotrabecular arrangement, resembling areas of HCC. Dotted line in **(H)** shows the massive infiltration of immune cells in one case of hepatic adenoma.

Histological examination of GHRLD livers with nodules showed that the nodule areas ranged from some macrovesicular steatosis with prominent steatosis and minor degree of nuclear pleomorphism, to others with more prominent nuclear variation including large cells with intranuclear inclusions (Figures 6A,B). The nodules were not distinctly separated from the adjacent liver by a capsule but showed distinct demarcation with some dilated vessels at the edges of the nodules (Figures 6C,D). The majority of the tumors represented adenomas similar to those of HNF1α-mutated type, though no mutations or stains have been attempted so far. In others, macrotrabecular arrangement was observed, indicating the development of HCC (Figures 6E–G) (20). In one case, the tumor had overwhelming lymphoid infiltrate and possibly represented an inflammatory adenoma (Figure 6H). Taken together, these results suggest that the NAFLD in GHRLD mice can spontaneously develop to hepatic adenomas, which have the potential to progress to HCC, even without any environmental insults.

**Figure 7:**
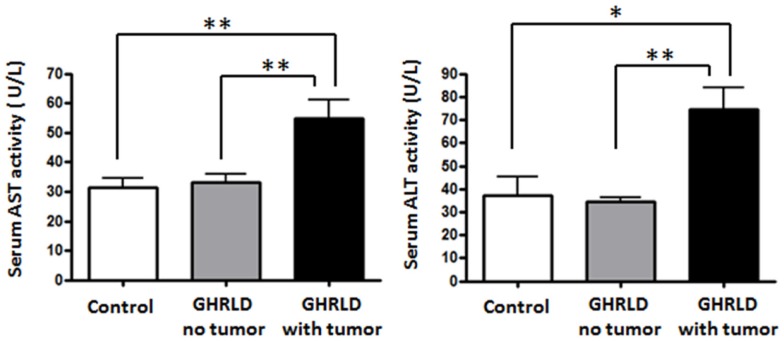
**Elevation of AST and ALT levels in sera of 50–70 weeks old GHRLD mice**. Serum samples of GHRLD mice with tumors (filled black bars, *n* = 5), GHRLD mice without tumors (filled gray bars, *n* = 6) and age-matched control mice (open bars, *n* = 5) were subjected to analyses of AST (left panel) and ALT (right panel) levels. *p < 0.05, **p < 0.01. No significant difference was observed between controls and GHRLD mice without hepatic tumor formation.

To further demonstrate the pathological impact of hepatic tumor formation on liver function, we examined the aspartate aminotransferase (AST) and alanine transaminase (ALT) activities in sera GHRLD mice (Figure 7). Both AST and ALT activities in GHRLD mice with hepatic tumors were significantly higher than those of controls. In contrast, no elevation of serum AST and ALT levels was observed in GHRLD mice with no obvious nodular structure formation in livers.

## Discussion

Obesity and metabolic syndrome have become major medical problems in developed countries (30) and affect children as well as adults (31, 32). One of the consequences of the metabolic disturbances in obesity is insulin resistance, resulting in excessive deposition of fat within liver, known as steatosis or NAFLD. In time, this NAFLD may progress to inflammatory changes with fibrosis as evidence for steatohepatitis, cirrhosis, and development of hepatocellular adenomas and carcinomas. While the molecular etiology of NAFLD is not fully understood, it can be considered as a hepatic manifestation of the metabolic syndrome. We and others (13–16) have previously shown that deletion of GH signaling in liver creates an insulin resistant state with compensatory hyperinsulinemia, disturbed glucose tolerance, and clinical hepatic steatosis from increased TG synthesis and decreased hepatic TG export. These disturbances persist in aged mice as evident in Figure 1. Rescue of GH signaling via adeno-viral restitution of the GHR in liver (13), or STAT5 signaling in cells (16) may reverse or prevent hepatic steatosis. Thus, deletion of GHR signaling in liver recapitulates the metabolic syndrome associated with obesity, which is itself a state of diminished GH secretion and hence diminished GH action on liver (33, 34). However, it should be pointed out that while hepatic GH signaling is compromised under the two conditions, the GH-deficient state is different: it is systemic in obese subjects, but is restricted to hepatocytes in GHRLD mice. In addition, GHRLD mice display elevated levels of circulating GH, which might have direct or indirect effects on lipid metabolism and insulin sensitivity in peripheral tissues (e.g., muscle and fat). Nevertheless, association between lower serum GH levels and NAFLD has been demonstrated in a recent cross sectional study (35); the GHRLD model could be used to unravel the contribution of compromised hepatic GH signaling in NAFLD and its progression.

Consistent with reports of progression in human obesity-associated NAFLD to fibrosis, steatohepatitis, and adenoma-carcinoma formation, we show in this study that evidence of inflammation and fibrosis is present in the livers of mice with liver-specific deletion of GH signaling. In some of these mice, the changes in livers progress to adenoma formation. Markers of fibrosis (Figure 4A) and inflammation (Figures 4B and 7) are markedly elevated. Our data suggest that abrogation of GH signaling in liver results in a metabolic profile consistent with that observed in the human metabolic syndrome, hepatic steatosis (NAFLD), which may progress to hepatic fibrosis, inflammation (NASH), and liver dysfunction (increased ALT/AST) eventually leading to hepatic adenoma formation. This sequence of events is similar to that of human obesity-related progression of insulin resistance, impaired glucose tolerance, hyperlipidemia, and liver abnormalities that include NAFLD, NASH, and rarely hepatic adenoma formation. Thus, our model may serve to evaluate the molecular mechanisms that define these transitions, determine which factors are responsible for progression to adenoma formation, and identify potential targets for treatment.

Our results are in line with previous findings on the roles of STAT5 in maintaining liver homeostasis. Similar to GHRLD, loss of STAT5 in liver tissue results in severe steatosis, progression to fibrosis (17), and eventual development of HCC at 17 months of age (36). The hepatic tumorigenesis can be accelerated with either carcinogen carbon tetrachloride (CCl4) treatment (37) or concurrent abrogation of the glucocorticoid (GC)-responsive glucocorticoid receptor (GR), another mediator of GH signaling in liver (38). Interestingly, 35% of STAT5/GR double-deficient mice display dysplastic nodules on livers and more than half of which progress to HCC at 12 months of age, in striking similarity to GHRLD males. Thus, impairment of the GH-STAT5/GR signaling cascade leads to severe defects in lipid homeostasis and spontaneous development of HCC.

Numerous factors could contribute to the tumorigenesis in aged GHRLD mice. Ccnd1, a member of the cyclin family, which is involved in cell-cycle G1/S transition and interactions with tumor suppressors, is overexpressed (Figure 3). Over expression of this gene may alter cell-cycle progression, has been observed in various tumors, and possibly contributed to tumor formation in our GHRLD mice (39). Similar to STAT5-deficient livers, hepatic transcription of tumor SOCS2 was also found decreased in GHRLD mice (40). Recently, Yu et al. showed recently that GH, through STAT5, regulates the expression of key proapoptotic proteins (PUMA and BIM) in liver, further highlighting the tumor suppressive function of the GH-STAT5 axis (36). Moreover, more evidence has accumulated implicating that hyperinsulinema and insulin-resistance, both present in GHRLD mice, could lead to deregulation of insulin receptor (IR), especially its IR-A isoform, in malignant cells and promote tumor progression (41, 42).

It has been suggested that inactivating mutations in HNF1α and activating mutations in β-catenin within the hepatocellular adenomas serve to classify clinical and prognostic factors such as progression to carcinoma as proposed for β catenin mutations (43–45). Obesity appears to amplify inflammation and other factors that promote tumorigenesis (46, 47). In our mouse model, it is not clear why only 30–40% of the animals develop the adenomas, and what, if any, molecular markers distinguish those animals that do from those that do not develop adenomas. These aspects require further research and could offer new insights into the pathophysiology of hepatic dysfunction in obesity, and the prevention of progression from NAFLD to tumor formation.

The world-wide epidemic of obesity has resulted in an increase of NAFLD in adults and children (4, 30–32). Because obesity is a state of diminished spontaneous and stimulated GH secretion (33, 34), it has been proposed that NAFLD in obesity has similar mechanisms to that observed in the animal models of decreased GH signaling in liver (13, 16). Hence, appropriately structured trials of treatment with GH have been proposed as a means of avoiding or diminishing the risk for NAFLD in obesity (16). If effective, such treatment would also diminish the risk of hepatic adenoma formation.

## Conflict of Interest Statement

The authors declare that the research was conducted in the absence of any commercial or financial relationships that could be construed as a potential conflict of interest.
